# Dr. Eleazar Parmly's Likeness

**Published:** 1842-12

**Authors:** 


*Dr. Eleazar Parmly's Likeness* —It was our intention, in accordance with a resolution of the American Society of Dental Surgeons, to have had Dr. Parmly's likeness in the first number of the present (third) volume of the Journal, to accompany and face the opening address, but we were unable to get it in time, consequently its appearance has been delayed until the publication of the second number. It should however be taken from this, when the Journal is bound, and placed in the fore part of the volume.

